# Nanoparticles Composed of Zn and ZnO Inhibit *Peronospora tabacina* Spore Germination *in vitro* and *P. tabacina* Infectivity on Tobacco Leaves

**DOI:** 10.3390/nano6030050

**Published:** 2016-03-16

**Authors:** George Wagner, Victor Korenkov, Jonathan D. Judy, Paul M. Bertsch

**Affiliations:** 1 Department of Plant and Soil Sciences, University of Kentucky, Lexington 40546, KY, USA; vdkore0@uky.edu (V.K.); jonathan.judy@csiro.au (J.D.J.); paul.bertsch@csiro.au (P.M.B.); 2 CSIRO Land and Water, Urrbrae 5064, Australia; 3 CSIRO Land and Water Flagship, CSIRO, Dutton Park 4102, Australia

**Keywords:** nanomaterials, nanopesticides, nanotechnology, nanotoxicity, nanotoxicology

## Abstract

Manufactured nanoparticles (NPs) are increasingly being used for commercial purposes and certain NP types have been shown to have broad spectrum antibacterial activity. In contrast, their activities against fungi and fungi-like oomycetes are less studied. Here, we examined the potential of two types of commercially available Zn NPs (Zn NPs and ZnO NPs) to inhibit spore germination and infectivity on tobacco leaves resulting from exposure to the fungi-like oomycete pathogen *Peronospora tabacina* (*P. tabacina*). Both types of NPs, as well as ZnCl_2_ and bulk ZnO control treatments, inhibited spore germination compared to a blank control. ZnO ENMs were shown to be a much more powerful suppressor of spore germination and infectivity than bulk ZnO. ZnO and Zn NPs significantly inhibited leaf infection at 8 and 10 mg·L^−1^, respectively. Both types of NPs were found to provide substantially higher concentration dependent inhibition of spore germination and infectivity than could be readily explained by the presence of dissolved Zn. These results suggest that both NP types have potential for use as economic, low-dose, potentially non-persistent anti-microbial agents against the oomycete *P. tabacina*.

## 1. Introduction

Transition metal compounds, particularly those containing Cu and Ag, have been used for centuries as antimicrobials [1]. Some ionic metal salts, e.g., of Zn and Cu, continue to be components of certain agrochemicals. Nanoparticles (NPs), including Zn NPs and ZnO NPs, have been shown to have antibacterial activity [2,3,4]. While still controversial, some recent studies indicate that metal-containing NPs can have higher antimicrobial activity to certain organisms than similar concentrations of the metal ions they contain [5,6,7,8,9,10,11,12]. The reasons for this enhancement are not clear, just as the mechanism(s) of nanoparticle (NP) uptake into cells and toxicity are not clear, even for the most studied forms [4,13]. Nevertheless, NPs may have potential as alternatives to organic pesticides if their efficacy and safety can be established.

Zn NPs may have several advantages over Ag and Cu NPs for use against plant pathogens in that Zn constitutes 0.004% of the earth’s crust and is an essential nutrient to plants and animals. Ionic Zn is therefore ubiquitous in the environment. Zn containing NPs are expected to dissolve to some degree under most conditions. Zn NPs and ZnO NPs, and their dissolution products, while being potentially toxic to plants and animals at high levels, may be tolerable in agricultural environments if doses required for pathogen control are low [6,14,15]. Indeed, ZnO is generally recognized as safe (GRAS) by the FDA and is used in food packaging and to fortify cereal-based foods.

Phytotoxicity of plants in the presence of high levels of ionic Zn, Zn NPs and ZnO NPs has been assessed in several studies by exposing seeds to NPs and observing impacts on seed germination and seedling growth [6,14,15,16]. Generally, concentrations in the range of 500 to 2000 mg·L^−1^ were shown to negatively impact germination and growth. For example, López-Moreno *et al.*, exposed soybean seeds to ZnO NPs at 0 to 4000 mg·L^−1^ [15]. Germination rate was not affected below 500 mg·L^−1^ but decreased above 2000 mg·L^−1^. In most of these studies, the potential impact of dissolved Zn in the experimental system was not assessed, but in the cases where it was assessed, NPs appeared to result in greater toxicity than when dissolved ionic Zn is present [6]. This is also evidenced from studies with bacteria and animal cells [17].

The phytoxicity of ZnO NPs has also been evaluated under field conditions in wheat plants grown in NP spiked soil in outdoor lysimeters [18]. TiO_2_ and ZnO NPs were mixed into soil at concentrations of 90.9 mg·kg^−1^ and 45 mg·kg^−1^, respectively, and after 2 months of aging, the soil was seeded with wheat plants. The authors found that after approximately 6 months wheat biomass had been reduced in the plants grown in the ZnO NP spiked lysimeters. However, the authors were unable to locate any ZnO NPs in the soil and speculated that all of the ZnO NPs had dissolved and that the reduction in biomass in the plants grown in the ZnO NP dosed lysimeters was the result of uptake of dissolved Zn. The conclusion from this study, namely that the ZnO NPs dissolved within 6 months in the soil, is important information in evaluating Zn NPs or ZnO NPs as potential pesticides. It suggests that routine application would not result in the persistent presence or accumulation of ZnO NPs within the soil, particularly where levels of NP applications are low.

*Peronospora tabacina* (*P. tabacina*) is a fungi-like oomycete pathogen related to the potato blight organism, *Peronospora infestans* (*P. infestans*), that infects tobacco and certain other members of the plant family, Solanaceae. Infection occurs after airborne spores contact leaves, germinate, and penetrate the plant epidermis with germ tubes. Hyphae grow throughout the leaf and form fruiting bodies, usually on the lower leaf epidermis, which produce spores to complete the life cycle and spread the infection. This disease, which can cause considerable crop loss, is currently controlled using the fungicide Metalaxyl. Resistance to this fungicide has been reported in *P. infestans* [19,20]. *P. tabacina* is an obligate biotroph, but there is an established, quantitative disease assay using tobacco plants [21] and simple methods are available for obtaining fresh, viable spores and for quantitative monitoring of germination.

We are not aware of reports of studies that assessed the impacts of applying Zn NPs or ZnO NPs onto leaves of mature plants to prevent/cure plant diseases, although the antifungal activity of ZnO NPs against the plant fungal pathogens *Botrytis cinerea* and *Penicillium expansum* has been studied *in vitro* [22]. It is unlikely that Zn NPs or ZnO NPs would easily penetrate the cuticle and incite phytotoxicity when sprayed in low concentration onto plant aerial surfaces, but this has not been assessed directly. Here, we have assessed the impacts of Zn NPs and ZnO NPs on *P. tabacina* spore germination and tobacco leaf infection in the laboratory and compared these effects to those observed as a result of exposure to ionic Zn and micron sized (bulk) ZnO, both of which can be components of some fungicide formulations.

## 2. Results and Discussion

### 2.1. Physical Properties of Zn Treatments

**Figure 1:**
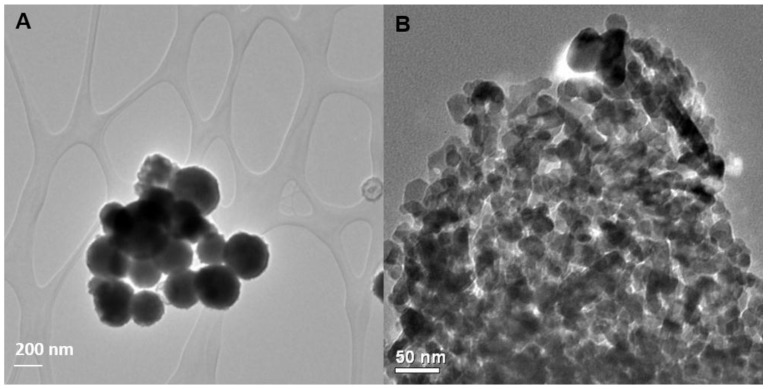
Transmission electron microscope (TEM) micrographs of (**A**) Zn nanoparticles (NPs) and (**B**) ZnO NPs.

Transmission electron microscope (TEM) measurements of the primary particle size of the Zn NPs revealed a mean particle diameter of 264 nm, much larger than the 50 nm indicated by the manufacturer and much larger than the 19.3 nm mean particle diameter measured for the ZnO NPs (Figure 1, Table 1). Variations such as these between manufacturers’ descriptions of NPs and their actual measured characteristics are not uncommon and emphasize the need for careful characterization of NPs purchased for experimental use. Measurements of the electrophoretic mobility of the NPs in deionized water (DI) indicated that the Zn NPs had a slightly negative surface charge of −1.6 mV and the ZnO NPs had a positive charge of 23.3 mV, the latter of which was comparable to the 12.5 mV zeta potential of the bulk ZnO treatment. Dynamic light scattering (DLS) measurements of NPs suspended in DI indicated that the Zn NPs and ZnO NPs had both formed large aggregates with mean diameters of 616 and 455 nm, respectively. These aggregates remain small relative to the particle size of the bulk ZnO treatment, for which the mean hydrodynamic diameter was 1886 nm.

Although settling of NP aggregates would affect many nanotoxicology exposures, the degree to which it might have affected germination and leaf-infectivity experiments made here is probably limited because during these assays NPs of both types and spores quickly settled in the droplets applied to glass slides (germination assay) or leaves (infectivity assay).

**Figure 2:**
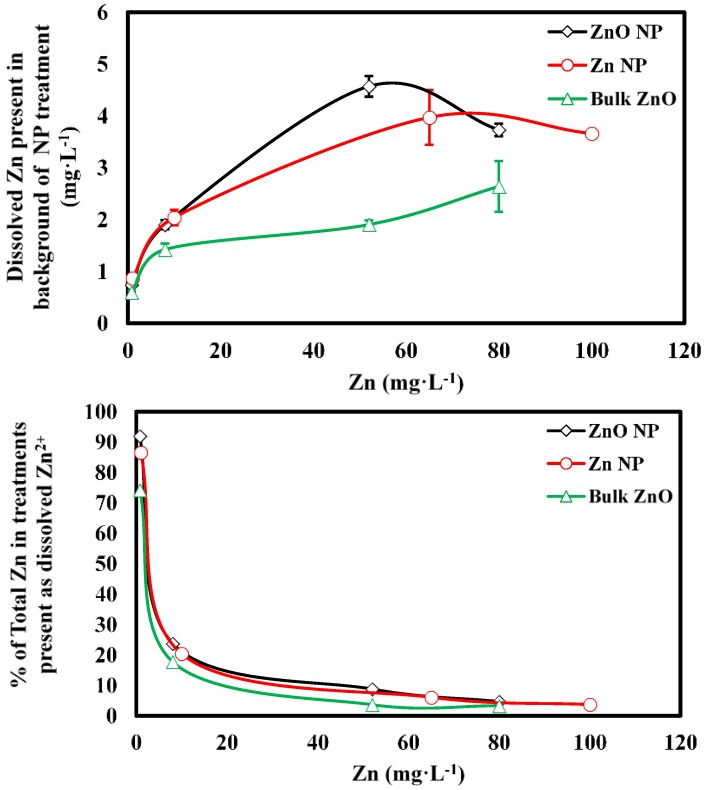
Dissolved Zn measured in the background of treatment suspensions (**top**) and the percentage of total Zn that is present at dissolved Zn (**bottom**).

Although ZnO is only sparingly soluble (maximum solubility of 1.6 mg·L^−1^ in cold water [23]) in water, some degree of dissolution of ZnO NPs occurs [24]. In our experimental system, the concentration of dissolved Zn in the background of each NP suspension indicated that at 1 mg·L^−1^ the Zn NPs and ZnO NPs were almost completely dissolved (Figure 2). Furthermore, as Zn concentration increases from 0 towards 100 mg·L^−1^ the concentration of dissolved Zn increases to 4–5 mg·L^−1^ but the proportion of Zn present in the dissolved phase decreases to <10% (Figure 2).

### 2.2. Impacts of Other Zn Treatments on Spore Germination

**Figure 3:**
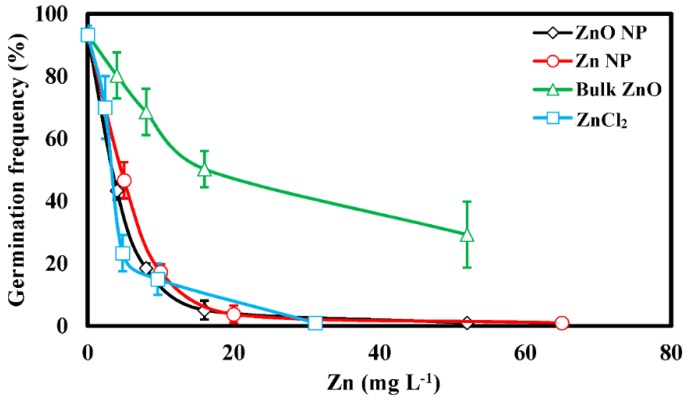
Germination frequency of *Peronospora tabacina* (*P. tabacina*) spores in the presence of Zn treatments. Error bars = standard deviation.

Control levels of spore germination were above 90% (0 mg·L^−1^, Figure 3). All four Zn treatments inhibited spore germination frequency, with Zn NPs, ZnO NPs, and ZnCl_2_ all inducing similar patterns of inhibition. In these treatments, germination was strongly affected at concentrations <10 mg·L^−1^ and almost completely ceased at 15–20 mg·L^−1^. However, the bulk ZnO treatment was much less inhibitory, with germination occurring for approximately 40% of spores at the highest concentration tested. Furthermore, the bulk ZnO treatment was significantly (α = 0.05) less inhibitory than the ZnO NP treatment at every concentration tested, indicating a fundamental difference in response resulting, at least indirectly, from the difference in particle size between these two treatments.

As previously mentioned, the highest amount of dissolved Zn estimated to be present in any of the Zn NP or ZnO NP treatments was approximately 5 mg·L^−1^ (Figure 2). However, at higher concentrations, the ZnO and Zn NPs were more inhibitory to spore germination than the 5 mg·L^−1^ ZnCl_2_ treatment. Therefore, the reduction in spore germination in the NP treatments was greater than could be explained by the concentration of dissolved Zn in the background of the NP suspensions. For example, 65 mg·L^−1^ Zn NP resulted in nearly 0% germination whereas 5 mg·L^−1^ Zn as ZnCl_2_ resulted in a significantly higher ~23% germination frequency (*p* = 0.034, Figure 3). The mechanism(s) responsible for this enhanced inhibition is (are) unclear. However, toxicity resulting from NP exposure in similar systems has been linked to reactive oxygen species generation as well as membrane disruption/disorganization [10,11,12,25]. Complex interactions of chloro-Zn complexes that impact fungal toxicity have also been observed and may be involved [26]. Furthermore, regarding the difference in germination between the bulk ZnO and Zn NP treatments, there was a smaller amount of dissolved Zn measured in the background of the bulk ZnO treatment. However, the difference in toxicity between the bulk ZnO and ZnO NP treatments is unlikely to be solely attributable to this difference, as the difference in dissolved Zn in these treatments is relatively small compared to the differences in toxicity.

**Figure 4:**
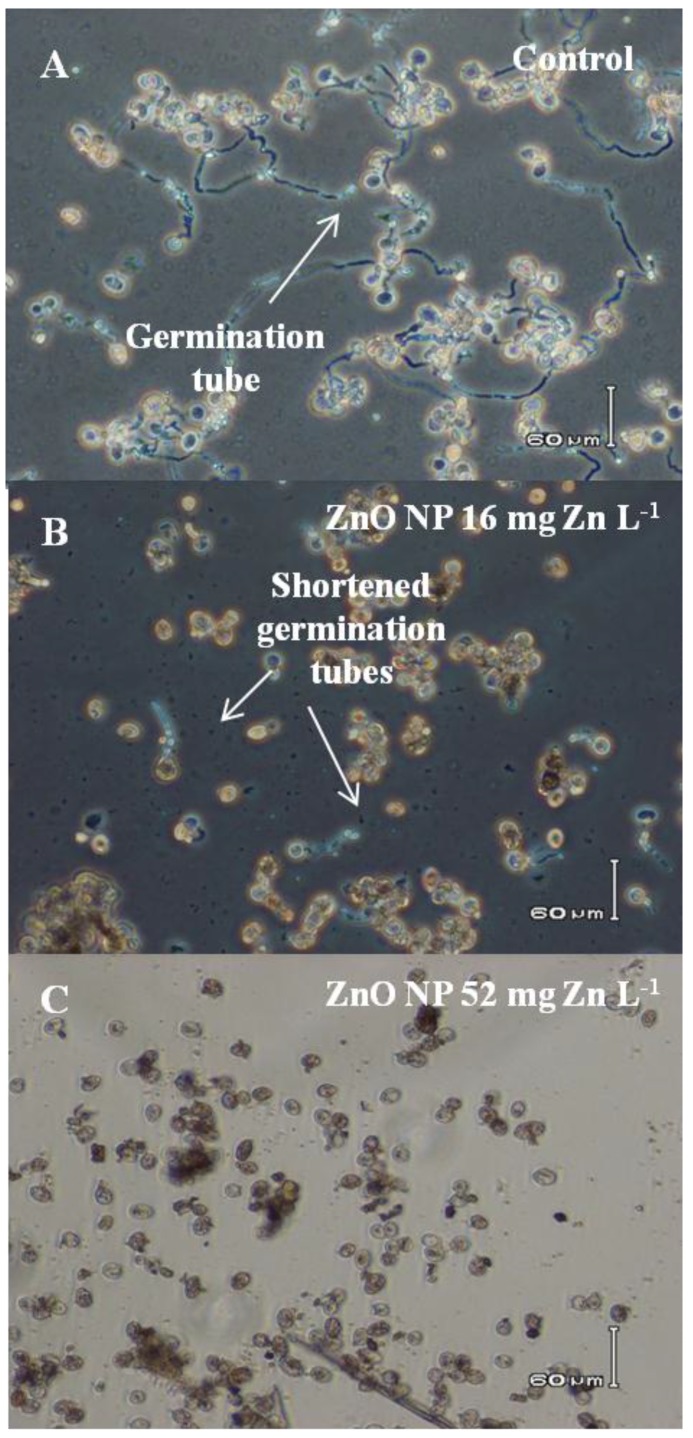
Effects on germination tube length in the presence of ZnO nanoparticles (NPs). Micrographs show spores germinated in water (**A**), in 16 mg·L^−1^ ZnO NPs (**B**), and 52 mg·L^−1^ ZnO NPs (**C**). Note the haloed appearance and long germination tubes of spores germinated in water *versus* the dark nature of most of those germinated in 16 mg·L^−1^ ZnO NPs, and all those germinated in 52 mg·L^−1^ ZnO NPs. Also note in Figure 4B that some spores had a haloed appearance but these only produced short germination tubes.

**Figure 5:**
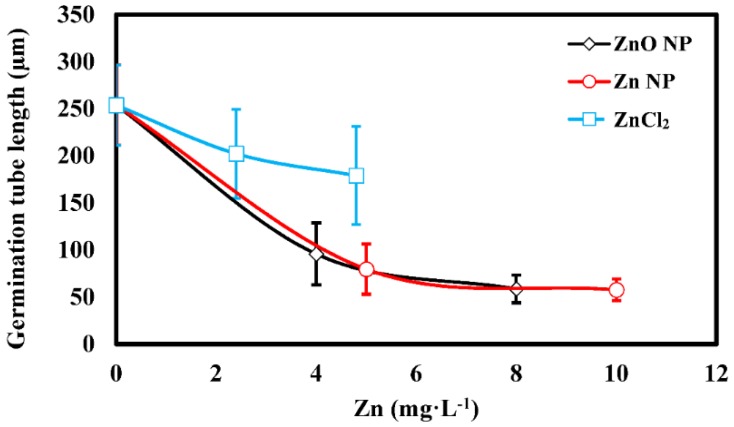
Spore germination tube length as measured for selected treatments. Error bars = standard deviation.

Microscopic examination of treated spores revealed that most control spores are bright in appearance and produce long germ tubes after incubation overnight in DI in the dark (Figure 4A). In contrast, many spores incubated with 16 mg·L^−1^ ZnO NPs (Figure 4B) were dark (dead) in appearance and only a few had short germ tubes (Figure 4B). Spores treated with 52 mg·L^−1^ ZnO NPs were dark in appearance and no germ tubes were observed (Figure 4C). Similar results were found for spores treated with Zn NPs. Measurement of germination tube elongation indicated a significantly greater reduction as a result of the NP treatments than was observed as a result of the ZnCl_2_ treatment (α = 0.05; Figure 5). In the NP treatments, germ tube elongation was reduced >60% at ≤5 mg·L^−1^, whereas germ tube length was only reduced 20.3% at 5 mg·L^−1^ by ZnCl_2_. These data are consistent with the inhibition observed in the germination tests (Figure 3). We speculate that inhibition of spores and disease potential (see below) occurs primarily at the stage of early germ tube growth, perhaps when young germ tube cell walls are most sensitive to penetration by NPs [27].

### 2.3. Impacts of NPs and Bulk Zn Forms on Infection

**Figure 6:**
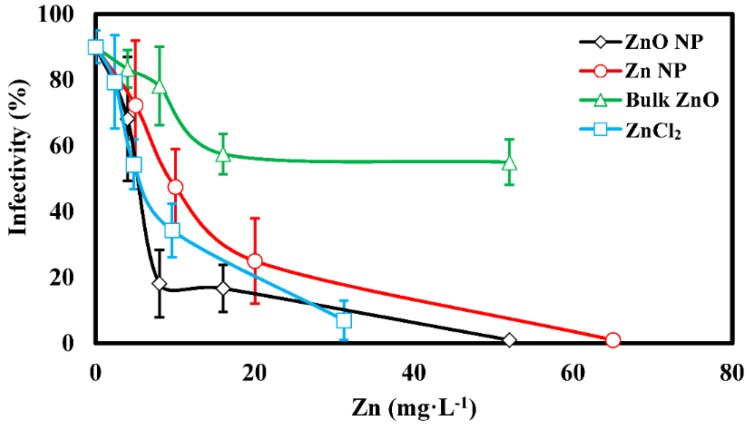
Percentage of tobacco plants infected by *P. tabacina* in the presence of Zn treatments. Error bars = standard deviation.

All four zinc treatments inhibited leaf infection, although the lowest concentration of each treatment did not result in a significant reduction compared to the control (Figure 6). This result is different from that from the spore germination tests, where all concentrations of each zinc treatment were significantly different than the control. Again using the 5 mg·L^−1^ ZnCl_2_ treatment to approximate effects attributable to Zn^2+^ in the background of the NP suspensions indicates that, starting at concentrations between 8 and 20 mg·L^−1^ for ZnO and Zn NP respectively, leaf infection was reduced to a significantly greater degree than in leaves treated with 5 mg·L^−1^ ZnCl_2_. This again suggests that the reductions in infection measured at these concentrations are not solely the result of the presence of Zn^2+^.

Compared to spore germination inhibition, a higher concentration of NPs was required to achieve a similar reduction in infection. This difference may be due to impacts of additional biochemicals present on leaf surfaces that are not present in germination tests, modifications of NPs on the plant surface, or other factors. Whatever the cause(s), this suggests that higher concentrations of NPs may be required to reach a desired level of disease control on the plant than is required to inhibit spore germination *in vitro*. Furthermore, conditions in the natural environment (field) as well as the conditions of agronomic application (e.g., suspension in tap water) may further reduce the effectiveness of these NPs. However, our results indicate that concentrations of the Zn NPs and ZnO NPs tested here are well below the levels likely to cause phytotoxicity (e.g., >500 mg·L^−1^), that were observed in the limited experiments reported in the literature. However, despite evidence that ZnO NPs will not be persistent in the environment [18], recent work suggests that ZnO NPs may pose a risk to sensitive plant-microbial relationships [28,29] and further study of the environmental implications of the agricultural application of ZnO NPs is required before field scale application could be responsibly undertaken.

## 3. Materials and Methods

### 3.1. Zn Treatments

Zn NPs, (Product #578002, 50 nm, > 99% trace metal basis) were obtained from Sigma-Aldrich (St. Louis, MO, USA) whereas ZnCl_2_ and bulk ZnO were purchased from Fisher Scientific (Fair Lawn, NJ, USA). ZnO NPs (Product #VK-J50, 50 ± 10 nm, >99.9% pure) were obtained from Xuancheng Jingrui New Materials Company (Xuan Cheng, China). Powders were mixed with DI at 22 °C, sonicated in a bath sonicator (FS3 Compact high performance ultrasonic cleaning system, Fisher Scientific, Fair Lawn, NJ, USA) operating at 22 W for 5 min and used within 6 h of preparation. Samples were vortexed just prior to their use.

### 3.2. Treatment Characterization

TEM size analyses were made using a Joel (JEOL Ltd., Akishima, Tokyo, Japan) 2010 TEM operating at 200 keV. Particle size was quantified based on measurements of at least 50 individual particles of each NP type from at least 3 different micrographs using ImageJ software Hydrodynamic diameter and electrophoretic mobility of NPs and bulk ZnO particles were measured on 100 mg·L^−1^ suspensions in deionized water using a Nano-ZS zetasizer (Malvern, UK). Electrophoretic mobility measurements were converted to zeta potential values using the Hückel approximation for the ENM treatments and the Smoluchowski approximation for the bulk ZnO treatment.

### 3.3. Measurement of Dissolved Zn in NP Preparations

For each NP type, a 100 mg·L^−1^ suspension in DI was sonicated in a bath sonicator for 2 min at 25 °C. Various dilutions were prepared in triplicate and incubated at 22 °C overnight in the dark before centrifugation for 1 h at 16,873× *g*. The top 250 µL of the supernatant below the meniscus was removed and diluted to 3 mL with deionized water and acidified to 0.15 M HNO_3_. Samples were analyzed for Zn content using an Agilent 7500cx ICP-MS (Santa Clara, CA, USA). Analytical runs contained spike recovery samples and analytical duplicate samples. Spike recovery was 108.2% ± 2.6% and mean relative difference between duplicate dilutions was 2.7% ± 2.5%.

### 3.4. P. Tabacina Spore Preparation

This oomycete pathogen is an obligate parasite that is easily maintained on living plants by infecting ~2 month old *Nicotiana tabacum* (variety Kentucky (KY)14) plants with *P. tabacina* (isolate KY79) spores as described in Reuveni *et al.* [30]. Freshly isolated, washed spores were diluted in DI to 100,000 spores mL^−1^ (counted with a hemocytometer) just prior to their use in germination or leaf-infection assays.

### 3.5 Spore Germination and Infectivity Tests

Spores were added to pH 7.5 solutions containing Zn NPs, ZnO NPs, ZnCl_2_, or bulk ZnO at concentrations ranging up to 65 mg·L^−1^ and tested for impacts on germination or on-leaf infectivity. These concentrations are relatively low compared to those sometimes used for foliar application of Zn (e.g., Zinc Sulfate 160 by Wilchem Ptd Ltd (Cavan, Australia)) is applied at up to 5 L·Ha^−1^ of 160 g·L^−1^) to correct micronutrient deficiency and would likely represent little environmental risk, though this would need to be confirmed. Germination tests were conducted on glass slides as described previously [21,31]. Briefly, spores (100,000 mL^−1^ in DI) were mixed with test solutions in DI and an 8 µL drop was placed on a glass microscope slide which was then incubated in a humid chamber at 22 °C in the dark for 16 h. Germination tube elongation was assessed for selected samples. For on-leaf infectivity tests, spores with or without test compounds in DI were applied as 4 µL drops to the upper epidermis of leaves of ~1.5 month old growth-chamber-grown KY14 tobacco plants (grown in 15 cm pots). Eight to ten individual drops were applied per leaf, near to and along the petiole [31]. Plants were incubated in humidified chambers for 16 h in the dark and then returned to the growth room. Plants were inspected for infection (yellow spots at treatment sites) after ~6 days. Water plus spores controls were applied to leaves on the same plant to assess maximum infection rate. Statistical analyses of germination and infectivity data were performed using SAS 9.4 (Cary, NC, USA). Treatment means were compared via pairwise one-sided exact Mann-Whitney *U*-tests.

## 4. Conclusions

In this work, we assessed the potential for Zn and ZnO NPs to control the fungi-like, oomycete pathogen *P. tabicina*. In summary, we show that two different Zn containing NPs can inhibit germination of spores of *P. tabacina* as well as inhibit disease on plants caused by this organism at relatively low concentrations. It is noteworthy that all forms of Zn were toxic to some degree.

To the best of our knowledge, this study is the first to investigate the effects of NPs on an oomycete, fungi-like pathogen, and the first to test the efficacy of NPs as a treatment to prevent infection by such a pathogen. Most reported studies involving NPs and plants have assessed phytotoxicity, and these have involved treating seeds or seedlings with high concentrations of NPs. Furthermore, our studies attempt to simulate conditions which may exist if the tested NPs were sprayed onto tobacco plants in an agricultural setting to prevent *P. tabacina* caused disease. We were also able to demonstrate that the toxicity observed in the oomycete spores was not simply the result of the presence of dissolved Zn in the NP preparations used and that the ZnO NP treatment was much more effective in controlling disease than bulk powder ZnO. Field studies are currently underway to determine the impacts of preventative spraying of *N. tabacum* with ZnCl_2_, ZnO NPs, and Zn NPs in the field on diseases caused by *P. tabacina* and other tobacco pathogens. Hypothetically, NPs may prove to be better retained on the leaf surface after spraying than water soluble Zn salts, resulting in more effective pest control. Furthermore, as the two different particle types differed in particle size, surface chemistry as well as in their composition, it is likely that systematic investigations examining the effect of particle characteristics could be conducted to optimize the effectiveness of Zn/ZnO NPs as an agent to control *P. tabicina* infection.

## Figures and Tables

**Table 1:** Treatment characterization data. Hydrodynamic diameter (Z-average diameter) measurements taken 2 min after bath sonication and based on intensity weighted size distribution measurements. SD = Standard deviation.

Treatment	Z-Average Diameter (nm)	Polydispersivity Index	TEM Diameter (Mean ± SD; nm)	TEM Range (nm)	Zeta Potential (mv ± Zeta Deviation)
Zn NP	615.8	0.57	263.5 ± 103.7	75.1–714.7	−1.6 ± 3.7
ZnO NP	453.3	0.58	19.3 ± 4.5	9.4–32.5	23.3 ± 5.0
Bulk ZnO	1886	0.45	N/A	N/A	12.5 ± 0.1

## Abbreviations

The following abbreviations are used in this manuscript:

DI: Deionized water
NP: Nanoparticle
TEM: Transmission electron microscope
DLS: Dynamic light scattering
